# Enhancement of Skin Permeability Prediction through PBPK Modeling, Bayesian Inference, and Experiment Design

**DOI:** 10.3390/pharmaceutics15122667

**Published:** 2023-11-24

**Authors:** Abdullah Hamadeh, Abdulkarim Najjar, John Troutman, Andrea Edginton

**Affiliations:** 1 School of Pharmacy, University of Waterloo, Kitchener, ON N2G 1C5, Canada; ahamadeh@uwaterloo.ca; 2 Systems In Silico Ltd., Waterloo, ON N2K 0B5, Canada; 3 Beiersdorf AG, 20245 Hamburg, Germany; abdulkarim.najjar@beiersdorf.com; 4 The Procter & Gamble Company, Mason, OH 45040, USA; troutman.ja@pg.com

**Keywords:** dermal, skin, modeling, pharmacokinetics, Bayesian, mechanistic, experiment design

## Abstract

Physiologically based pharmacokinetic (PBPK) models of skin absorption are a powerful resource for estimating drug delivery and chemical risk of dermatological products. This paper presents a PBPK workflow for the quantification of the mechanistic determinants of skin permeability and the use of these quantities in the prediction of skin absorption in novel contexts. A state-of-the-art mechanistic model of dermal absorption was programmed into an open-source modeling framework. A sensitivity analysis was performed to identify the uncertain compound-specific, individual-specific, and site-specific model parameters that impact permeability. A Bayesian Markov Chain Monte Carlo algorithm was employed to derive distributions of these parameters given in vitro experimental permeability measurements. Extrapolations to novel contexts were generated by simulating the model following its update with samples drawn from the learned distributions as well as parameters that represent the intended scenario. This algorithm was applied multiple times, each using a unique set of permeability measurements sourced under experimental contexts that differ in terms of the compound, vehicle pH, skin sample anatomical site, and the number of compounds under which each subject’s skin samples were tested. Among the data sets used in this study, the highest accuracy and precision in the extrapolated permeability was achieved in those that include measurements conducted under multiple vehicle pH levels and in which individual subjects’ skin samples are tested under multiple compounds. This work thus identifies factors for consideration in the design of experiments for the purpose of training dermal models to robustly estimate drug delivery and chemical risk.

## 1. Introduction

The development of dermatological drugs and skin products necessitates an in-depth understanding of systemic exposure to xenobiotics present in these formulations. Measuring this exposure typically involves in vitro [1] or in vivo testing of these products [2,3,4]. However, such experimental approaches have their limitations as they primarily provide insights into dermal penetration under specific conditions, often failing to account for the variations stemming from inter-individual differences, intra-individual differences between anatomical sites of application, or diversity in application scenarios. Furthermore, these experiments can be resource-intensive and time-consuming.

To address these challenges, predictive models of dermal absorption can serve as a powerful tool in the evaluation of targeted formulation design and assessing chemical risks associated with topically applied products [5]. One class of predictive models, based on empirical Quantitative Structure–Property Relationships (QSPRs), predict skin permeability based on correlations that are functions of characteristics of the permeating compound, such as molecular weight and lipophilicity [6,7,8] or other descriptors that are derived via computational methods [9].

Another class of models derives from mechanistic considerations of the physical processes underpinning solute absorption and transdermal diffusion. Such physiologically based pharmacokinetic (PBPK) dermal absorption models capture the spatial structure of the dermal membrane as well as the kinetics of solute transport and metabolism within the skin. They additionally encompass a diverse set of parameters, ranging from the properties of the permeating chemical, the vehicle, the application method, and environmental factors, to the characteristics of the skin itself.

PBPK models offer versatility in predicting the dermal disposition of chemicals in highly diverse and untested scenarios, thus reducing the need for extensive experimental studies. However, these models are often limited by parameter uncertainty as well as unknown levels of variability in model quantities. These sources of uncertainty and variability can ultimately be reflected in widened confidence intervals in measures of dermal disposition.

Prior to this study, the PBPK skin permeation model by Dancik et al. [10] was adapted into MoBi and made available on GitHub [11]. This model consists of a one-dimensional partial differential equation, but it lacks a pathway for ionized or polar species, leading to under-prediction of hydrophilic compound absorption. Two modifications were proposed to address this issue in later work by Kasting et al. and Yu et al. [12,13], which were the addition of a follicular pathway bypassing the lipophilic environment of the stratum corneum, and the addition of a transcellular pathway for polar species via pores within the stratum corneum. Besides extending the earlier model in Dancik et al. [10] to polar molecules, the Kasting et al. and Yu et al. [12,13] models are parameterized to describe the anatomy of these additional pathways. This makes it possible to tune the skin descriptors in these pathways to represent the properties of skin in distinct body regions. These include quantities such as follicle density, hair shaft diameter, and follicle orifice diameter, which are known to vary across anatomical sites [14,15,16,17,18]. Extensive validation of this model was performed in Kasting et al. and Yu et al. [12,13] against permeability measurements reported in Wang et al., 2007 [19], Chen et al., 2013 [20], and Baba et al., 2017 [9]. In this study, the MoBi implementation of the Dancik et al. [10] model was updated to include both of these additional pathways, and model details are provided in Nomenclature and Appendix A.

**Figure 1:**
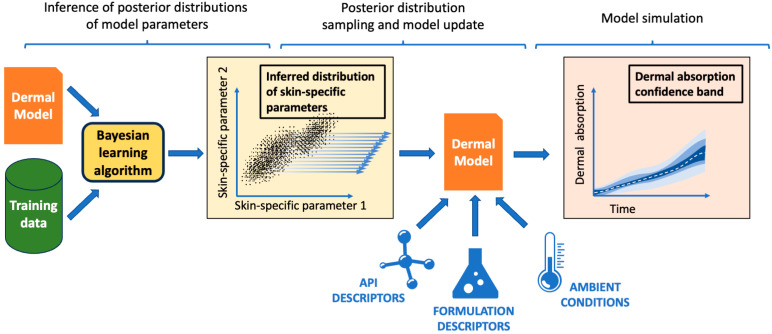
Illustration of the model training and extrapolation workflow from Hamadeh et al. [21]. Joint posterior distributions of model parameters are inferred from experimental training data. The trained model is then extrapolated to estimate dermal disposition in application scenarios of interest: first, the inferred distributions are repeatedly sampled. Next, these samples are combined with descriptors of the active pharmaceutical ingredient (API), the formulation, and ambient conditions to update the dermal model. The updated model is subsequently simulated for each sampled parameter, resulting in a range of dermal disposition estimates.

In Hamadeh et al. [21], a Bayesian learning and extrapolation algorithm was employed to train PBPK dermal models. As depicted in the example workflow in Figure 1, this algorithm infers the variability and uncertainty in dermal model parameters from experimental training data, such as in vitro permeation tests. The training process yields nonparametric joint probability distributions for these parameters, capturing their ranges and correlations. These inferred distributions can then inform predictions beyond the scope of the original training data. This extrapolation process involves updating and then simulating the dermal model with samples drawn from the inferred distributions of model parameters, known properties of the permeating active pharmaceutical ingredient (API) of interest, the formulation, and ambient application conditions. Simulation of the updated model using numerous samples from the inferred distributions yields predictions of the expected range of dermal absorption under the intended scenario.

The experimental contexts from which training data are sourced can differ in terms of the selections and diversities of test compounds, vehicles, subjects, anatomical sites, experiment protocols, and ambient conditions. In addition, the identifiability of model parameters can strongly depend on the choice of measurements used to train the model. For instance, when measuring the permeability of multiple compounds across the skin of a single individual, any observed disparities in permeability between the compounds can be attributed to distinct physical and chemical properties of the permeants, rather than the individual’s skin characteristics. Similarly, experiments that measure the permeability of ionizable compounds while controlling all variables except for the vehicle pH allow the learning algorithm to distinguish between the characteristics of both polar and non-polar permeation pathways.

This study examines how the choice of training data, from which model parameter distributions are inferred, influences the trained model’s accuracy and precision in predicting the skin permeability coefficient in previously untested dermal application scenarios. A standard workflow, based on the learning algorithm of Hamadeh et al. [21], is adopted to separately train the model presented in Appendix A using each one of a series of data sets. Next, the model’s extrapolative performance is assessed in experimental contexts that differ from those of the training data in terms of the individuals to whom the drug is applied, the vehicle pH, the anatomical site, and the permeating compound. Finally, a comparison of the inferred parameter distributions is conducted to assess the impact of training data on the identifiability of the permeability coefficients across the skin via polar and non-polar routes.

## 2. Methods

### Data

Roy and Flynn, 1990 [22], reported the results of diffusion cell measurements of fentanyl and sufentanil permeability across human cadaver skin samples. In these experiments, skin samples with an area of 0.785 cm^2^ were clamped between 3 mL donor and receptor chambers filled by citrate phosphate buffers of varying pH levels. The permeant flux penetrating the skin was allowed to reach steady-state conditions, and skin permeability was measured from the steady-state flux profile.

**Figure 2:**
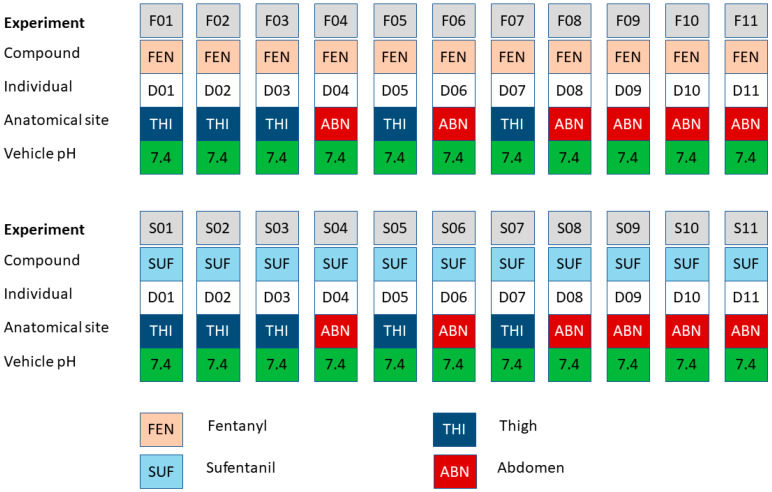
Combinations of permeants, individuals, anatomical sites, and vehicle pH levels used in the pH 7.4 experiments, the results of which are summarized in Table 1, as reported in Roy and Flynn, 1990 [22].

In one set of experiments, the results of which are summarized in Table 1, permeability across heat-separated epidermis samples from each of eleven skin donors (D01 to D11) was measured for both fentanyl (experiments F01 to F11) and sufentanil (experiments S01 to S11). Each donor contributed sections from either the thigh or abdominal regions. The permeant concentration in the donor solution was set to saturation and held at pH 7.4. The combinations of skin donors, permeant compounds, and anatomical sites used in the various experiments are illustrated in Figure 2.

**Figure 3:**
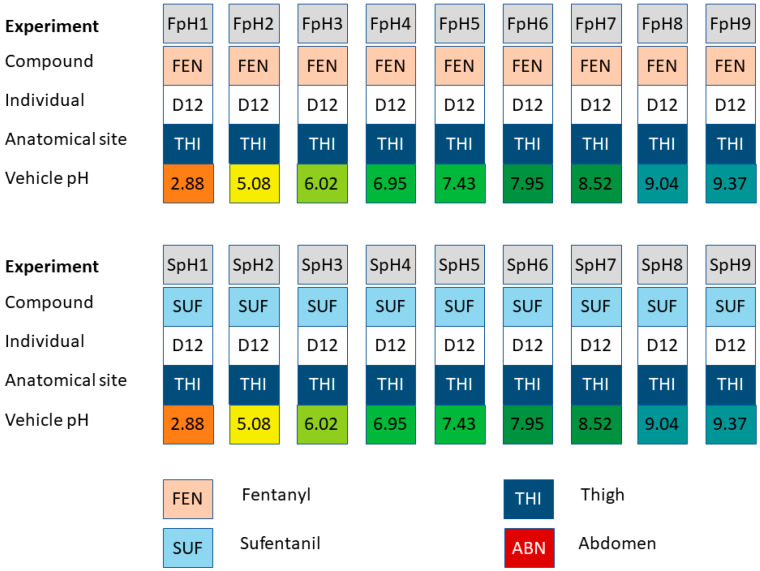
Combinations of permeants, anatomical sites, and vehicle pH levels used in the variable pH experiments by Roy and Flynn, 1990 [22]. All experiments in this set were conducted using skin samples sourced from a single donor (D12). The corresponding permeability measurements are summarized in Table 2.

A second set of experiments reported in Roy and Flynn, 1990 [22], tested fentanyl (experiments FpH1 to FpH9) and sufentanil (experiments SpH1 to SpH9) permeability across dermatomed thigh skin sections at nine different pH levels, from pH 2.88 to 9.37 (Table 2). The skin sections in this second set were sourced from a single individual (D12). This combination of experiments is illustrated in Figure 3.

## 3. Experiment Data Set Combinations for Model Training

**Figure 4:**
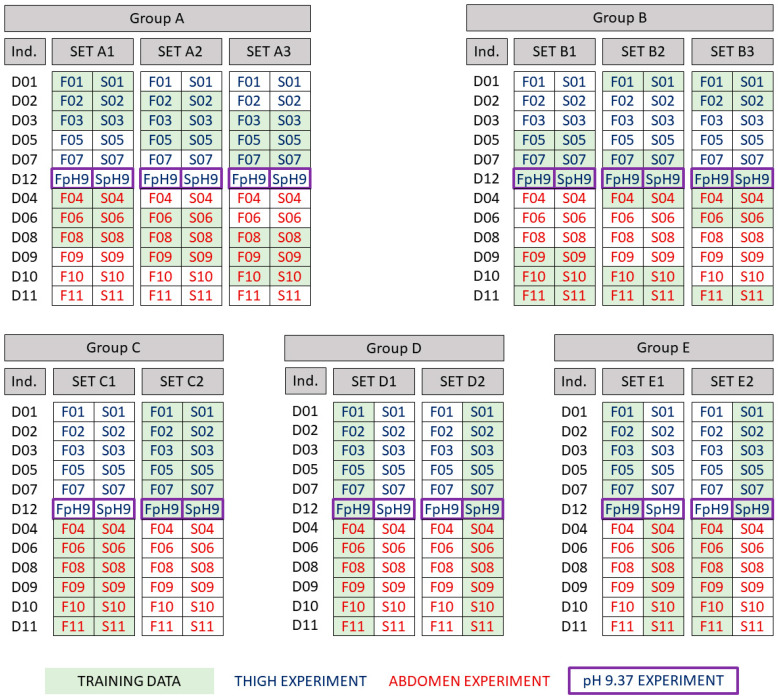
Groups of data sets used in model training.

A selection of the measurements in Table 1 and Table 2 were arranged into five groups of two or three data sets with which to independently train the model. We denote these groups as A, B, C, D, and E. Each data set within these groups consisted of a combination of twelve experiments, as shown in Figure 4. The groups differed in terms of the experimental contexts of the twelve experiments:

- **Group A: Cross-over design, common pH level.** Group A encompasses three distinct data sets, each containing permeability measurements for skin samples obtained from six individuals. Three individuals contributed thigh samples, and three contributed abdominal samples. These data sets adopted a cross-over experimental design, exposing each individual’s skin to both fentanyl and sufentanil. All skin samples were tested using pH 7.4 vehicles.
- **Group B: Cross-over design, varying pH levels.** Group B encompasses three distinct data sets, each containing permeability measurements for skin samples obtained from six individuals. Three individuals contributed thigh samples, and three contributed abdominal samples. These data sets adopted a cross-over experimental design, exposing each individual’s skin to both fentanyl and sufentanil. In each data set, one individual’s skin samples were tested using a pH 9.37 vehicle, while the samples from the remaining five individuals were tested at pH 7.4.
- **Group C: Cross-over design, single anatomical site per data set.** Group C consisted of two data sets, one of which used only abdominal skin samples from six individuals, and the other only thigh skin samples from a different set of six individuals. The latter data set included fentanyl and sufentanil experiments from a single individual conducted using a pH 9.37 vehicle, with all remaining samples tested at pH 7.4.
- **Group D: Single compound per data set.** Group D consisted of two data sets. Each data set consisted of measurements of skin permeability of only one compound, either fentanyl or sufentanil, across skin samples from twelve individuals. Both data sets included permeability measurements across thigh skin from one individual conducted using a pH 9.37 vehicle, with all remaining samples tested at pH 7.4.
- **Group E: Parallel design, varying anatomical site, varying pH levels.** Group E consisted of two data sets. The first data set comprised permeability measurements conducted using six thigh samples from six individuals that were tested with fentanyl, and six abdominal samples from another six individuals that were tested with sufentanil. The second data set comprised the inverse combination: six thigh samples were tested with sufentanil, and six abdominal samples were tested with fentanyl. In both data sets, among the thigh samples, one donor sample was tested using a pH 9.37 vehicle.

## 4. Model Parameters and Notation

We classify the parameters of interest in the model as being specific to one of three model components: the compound ($k_{C}$), the anatomical site ($k_{S}$), and the individual ($k_{I}$). Nominal values for these quantities are provided in the original models by Kasting et al. [12] and Yu et al. [13], which are summarized in the model in Appendix A. We use the double subscript notation $k_{C_{fen}}$, $k_{C_{suf}}$ to denote the nominal values of $k_{C}$ for fentanyl and sufentanil, respectively. Similarly, $k_{S_{abd}}$, $k_{S_{thi}}$ denote the literature-derived nominal values of $k_{S}$ for abdominal and thigh skin.

We introduce into this model a set of uncertain parameters, $\theta_{C}$, $\theta_{S}$, $\theta_{I}$, that scale the values of nominal parameters $k_{C}$, $k_{S}$, $k_{I}$, respectively. We denote the model-generated estimate of permeability $P_{tot/w}$ across a dermal section by the function $P_{tot/w}=f\left(\theta_{C}k_{C},\theta_{S}k_{S},\theta_{I}k_{I}\right)$. Since $\theta_{C},\theta_{S},\theta_{I}$ are scaling parameters, $f\left(k_{C},k_{S},k_{I}\right)$ recovers the permeability for an average individual based on the nominal parameter values in [12,13].

We denote by $p\left(\theta_{C}\right)$, $p\left(\theta_{S}\right)$, $p\left(\theta_{I}\right)$ the parameters’ respective literature-derived prior distributions.

## 5. Bayesian Learning of Inter-Individual and Inter-Site Variability

We next describe a Bayesian Markov Chain Monte Carlo (MCMC) learning procedure to infer the joint probability distributions of the model scaling parameters $\theta_{C},\theta_{S},\theta_{I}$ given the dermal permeability measurements taken from one of the data sets within Groups A–E (Figure 4). Each data set is a unique collection of permeability measurements that vary in terms of the permeating compound, the anatomical site from which the skin sample is taken, the individual from whom the skin sample is sourced, and the vehicle pH. We denote a single data set from among those in Groups A–E by $d_{C,S,I}$. Here, the subscripts represent compounds included within the data set (C), the anatomical sites (S) of the skin samples tested with the data set, and the collection of individuals (I) from whom the skin samples tested within the data set were sourced. We also assume, without a loss of generality, that these experimental measurements are obtained through a common experimental protocol that is subject to a common relative measurement error variance of $\nu^{2}$ that has a prior distribution $p\left(\nu^{2}\right)$.

We let $p\left(\theta_{C},\theta_{S},\theta_{I}\right)$ denote the joint distribution of the priors on $\theta_{C},\theta_{S},\theta_{I}$. The posterior distribution of these parameters given the observations $d_{C,S,I}$ is

(1) $$p\left(\theta_{C},\theta_{S},\theta_{I},\nu |d_{C,S,I}\right)\propto L\left(\theta_{C},\theta_{S},\theta_{I},\nu |d_{C,S,I}\right)\cdot p\left(\theta_{C},\theta_{S},\theta_{I}\right)\cdot p\left(\nu^{2}\right)$$

where

(2) $$L\left(\theta_{C},\theta_{S},\theta_{I},\nu |d_{C,S,I}\right)=\prod_{C,S,I}\frac{1}{\sqrt{2\pi \nu^{2}}}\exp\frac{-{\left(\log\left(f\left({\theta_{C}k}_{C},{\theta_{S}k}_{S},\theta_{I}k_{I}\right)\right)-\log\left(d_{C,S,I}\right)\right)}^{2}}{2\nu^{2}}\;\;\;$$

is the likelihood of the observations $d_{C,S,I}$ given $\theta_{C},\theta_{S},\theta_{I},\nu$. The prior distributions for scaling parameters $\theta_{C},\theta_{S},\theta_{I}$ are derived from the literature or otherwise assumed to be non-informative. The measurement error variance is assumed to be log-uniformly distributed, and, as such, we have $p\left(\nu^{2}\right)\propto 1/\nu^{2}$.

A parallelizable, adaptive, block Metropolis–Hastings algorithm based on the procedure in Hamadeh et al. [21] was developed in the ospsuite-R package (v11) and utilized to obtain samples from the joint posterior distribution $p\left(\theta_{C},\theta_{S},\theta_{I},\nu |d_{C,S,I}\right)$.

## 6. Selection of Model Parameters for Inference

To select the model parameters to be inferred based on experimental data, we first conducted a literature review to identify the ranges of the uncertain model parameters. Sensitivity analyses based on the Morris method [23] were then conducted to assess the potential of the uncertain skin-specific model to affect the permeability of fentanyl and sufentanil at vehicle pH levels 7.4 and 9.37, which correspond to the pH levels of the vehicles used in the experiments in the training data sets. The Morris method is a global sensitivity method in which the impact of each parameter on the output of interest (the permeability of fentanyl or sufentanil) is assessed at multiple points in the parameter space while varying the remaining parameters. This allows for a more holistic quantification of the sensitivity of the output to a given parameter than a local sensitivity analysis. The results of the Morris method consist of an average sensitivity measure (µ*) and a standard deviation of the sensitivity (σ). Whereas µ* indicates the direct impact of a parameter on the model output, σ measures the degree to which the parameter’s impact depends on the values of other uncertain parameters.

## 7. Internal Validation

Internal validations of the inferred posterior distributions were conducted as follows: for each measurement in a given training data set $d_{C,S,I}$, the dermal model was first updated with nominal parameter values $k_{C},k_{S},k_{I}$ that reflect the experimental conditions from which that measurement was taken. Subsequently, the model was simulated repeatedly using parameter samples drawn from the inferred joint posterior distribution $p\left(\theta_{C},\theta_{S},\theta_{I},\nu |d_{C,S,I}\right)$ for the data set, and the corresponding permeability $f\left({\theta_{C}k}_{C},{\theta_{S}k}_{S},\theta_{I}k_{I}\right)$ was evaluated for each sample. A visual predictive check was generated to compare the distribution of these resulting estimates against their corresponding permeability measurements.

## 8. External Validation

### 8.1. External Validation 1

In this first external validation, predictions of skin permeability based on the inferred posterior distribution $p\left(\theta_{C},\theta_{S},\theta_{I}|d_{C,S,I}\right)$ for each data set $d_{C,S,I}$ were generated under each of the following four scenarios: (1) fentanyl applied to abdominal skin, (2) fentanyl applied to thigh skin, (3) sufentanil applied to abdominal skin, (4) sufentanil applied to thigh skin. A pH 7.4 vehicle was assumed, representing the conditions of the experiments summarized in Table 1. For each of the four combinations of compound and anatomical site, the dermal model was first updated with the corresponding nominal parameter values $k_{C}$ and $k_{S}$ as well as average values for individual-specific parameters $k_{I}$ from [12,13]. Under each of the four scenarios, and for each data set $d_{C,S,I}$ in Groups A–E, the inferred joint posterior distribution $p\left(\theta_{C},\theta_{S},\theta_{I}|d_{C,S,I}\right)$ was sampled to obtained draws of the parameters $\theta_{C},\theta_{S},\theta_{I}$ corresponding to the compound and anatomical site pair being simulated and corresponding to each individual in the data set $d_{C,S,I}$. Using these sampled parameter values, the permeability $f\left({\theta_{C}k}_{C},{\theta_{S}k}_{S},\theta_{I}k_{I}\right)$ was predicted by the model for each individual in the data set $d_{C,S,I}$.

In Groups C and D, each data set contained measurements from only one specific anatomical site or compound, respectively. However, the external validation under scenarios 1–4 requires predicting permeability for anatomical sites or compounds both within and outside the data sets in Groups C and D. Nevertheless, as shown in Hamadeh et al. [21] (Supplementary Material), it is reasonable to assume that the correlations existing between compound-specific and skin-specific parameters are similar across compounds. For this reason, we substitute the inferred scaling parameters that are specific to one compound or anatomical site when extrapolating the model to another compound or anatomical site, respectively.

As an example, the posterior distribution inferred from data set D1 did not include any sufentanil-specific parameters since the D1 data set experiments only measured fentanyl permeability. However, we assume here that the correlations between scaling parameters $\theta_{C_{fen}},\theta_{S},\theta_{I}$ that were inferred from data set D1 approximate the correlations between scaling parameters $\theta_{C_{suf}},\theta_{S},\theta_{I}$. Therefore, to approximate the permeability of sufentanil across thigh skin, we evaluate the permeability $f\left(\theta_{C_{fen}}{k_{C}}_{suf},\theta_{S_{thi}}{k_{S}}_{thi},\theta_{I_{k}}k_{I}\right)$ using samples $\left(\theta_{C_{fen}},\theta_{S_{thi}},\theta_{I}\right)$ drawn from the posterior distribution $p\left(\theta_{C_{fen}},\theta_{S_{thi}},\theta_{I_{k}}|d_{C,S,I}=D1\right)$.

### 8.2. External Validation 2

In the second external validation, predictions of permeability were generated based on the inferred posterior distribution $p\left(\theta_{C},\theta_{S},\theta_{I}|d_{C,S,I}\right)$ for each data set $d_{C,S,I}$ under the conditions of the experiments in Table 2. These experiments involved the application of fentanyl and sufentanil to thigh skin sourced from individual D12 and spanned a vehicle pH range of 2.88 to 9.04. For each experiment, the model was updated with nominal parameters $k_{C}$, $k_{S}$, and $k_{I}$, as well as the corresponding vehicle pH. Subsequently, the model was extrapolated to predict permeability by iteratively sampling parameters $\theta_{C},\theta_{S},\theta_{I}$ from the posterior distribution and evaluating the permeability estimate $f\left({\theta_{C}k}_{C},{\theta_{S}k}_{S},\theta_{I}k_{I}\right)$. These permeability estimates were then compared to the corresponding measurements in Table 2. Here, parameters $\theta_{I}$ specific to individual D12 were used when validating the posteriors inferred from data sets $d_{C,S,I}$ in Groups B–E since these groups included permeability measurements for that donor at vehicle pH 9.37. Since data sets A1–A3 did not include measurements for donor D12, parameters $\theta_{I}$ were instead sampled from all donors in those respective data sets.

## 9. Analysis of Pathway-Specific Permeability

As discussed in [12,13] and in Appendix A, the aggregate permeability $P_{tot/w}$ is composed of contributions from an appendageal pathway, a non-polar transcellular pathway via stratum corneum lipids, and a polar pathway via micropores present in the stratum corneum lipid matrix.

Following the inference and validation steps above, the joint posterior distributions of compound-, site-, and individual-specific parameters were iteratively sampled. These samples were subsequently employed to compute the permeabilities of both the appendageal and non-polar transcellular pathways, yielding nonparametric distributions of the permeability across each pathway.

## 10. Results

### Literature Review of Parameter Uncertainties

The chemical properties of relevance for fentanyl and sufentanil are given in Table 3. As previously reported in [21], uncertainty in compound-specific parameters resides primarily in the trans-bilayer permeability ($k_{trans}$) and the partition coefficient between the SC lipid phase and water ($K_{lip/w}$). These parameters are QSPRs that are functions of the molecular weight and of the lipophilicity values given in Table 3. Two more model parameters that depend on compound-specific quantities are the partition coefficient of the permeant in the infundibulum relative to water ($K_{inf/w}$) and the diffusivity of the permeant in the infundibulum $D_{inf}$. It is proposed in Yu et al. [13] that in the in vitro context, the infundibulum is filled with a vehicle-like fluid, and therefore, the nominal values of these parameters are equal to the nominal values of the partition and diffusion coefficients, respectively, in the aqueous vehicle. The partition coefficient in the vehicle, and therefore $K_{inf/w}$, has as a nominal value that is given by the inverse of the permeant’s non-ionized fraction in water. On the other hand, $D_{inf}$ is given by the aqueous diffusivity formula in Yu et al. [13]. An uncertainty of ±1 log_10_ unit is assumed for the infundibulum parameters. The nominal values and the uncertainties for these compound-specific quantities are given in Table 4.

**Table 3:** Fixed values for physical/chemical properties of permeants.

Quantity (Units)	Fentanyl	Sufentanil	Reference
Molecular weight (MW) (g/mol)	336.5	386.5	[24]
$pK_{a}$ (basic)	8.99	8.56	[22]
$\log_{10}K_{o/w}$ (octanol/water)	2.86	3.45	[24]

**Table 4:** Uncertainty ranges for QSPRs of compound-specific parameters of the skin permeation model. See Nomenclature.

Parameter (Units)	Description	Value	Reference
$\log_{10}k_{trans}$ (cm/s)	Trans-lipid bilayer permeability in a hydrated stratum corneum.	$\mathrm{Nominal}\;\mathrm{value}:-0.725-0.792MW^{\frac{1}{3}}$ Uncertainty range: Nominal value ± 1.08	[19]
$\log_{10}K_{lip/w\;}$	Partition coefficient of permeant in SC lipids with respect to water.	$\mathrm{Nominal}\;\mathrm{value}:\;0.81\log_{10}K_{o/w}+\log_{10}0.43$ Uncertainty range: Nominal value ± 0.434	[25,26]
$\log_{10}K_{inf/w\;}$	Partition coefficient of permeant in infundibulum with respect to water, assuming aqueous.	Nominal value: $\log_{10}\left(\frac{1}{f_{non/water}}\right)=\log_{10}\left(1+10^{pK_{a}-pH}\right)$ Uncertainty range: Nominal value ± 1	[13]
$\log_{10}D_{inf\;}$(cm^2^/s)	Diffusion coefficient of permeant in infundibulum with respect to water.	Nominal value (aqueous vehicles): $A_{D_{aq}}+B_{D_{aq}}MW$, for constants $A_{D_{aq}},B_{D_{aq}}$ as defined in equation T1_4, Supplementary Material, ref. [13] for the aqueous diffusivity $D_{aq}$. Uncertainty range: Nominal value ± 1	[13]

Notation: MW : permeant molecular weight. $K_{o/w}$ : permeant lipophilicity.

Values of fixed skin-specific quantities used in the dermal model are given in Table 5. Table 6 presents literature-derived nominal values and, where available, the ranges, for the uncertain skin-specific parameters of the dermal model detailed in Appendix A. The justification for these parameters is as follows:

- Stratum corneum thickness: Table 6 in [27] shows inter-site and within-site variability. The abdominal $h_{SC}$ varies between 6 and 13 µm for a partially hydrated SC. For a fully hydrated SC, reference [10] proposes a stratum corneum thickness of 43 µm. To capture the variability in Table 6 in [27], we applied a similar uncertainty to the fully hydrated case.
- The range of the combined thickness $m_{y}$ of the stratum corneum lipid bilayer envelope and corneocyte thickness was calculated based on the number of cell layers in the stratum corneum reported in [28].
- Reference [12] proposes a nominal follicle density $N_{f}$ of 24/cm^2^. However, reference [16] reports inter-region variability in follicle density that ranges between 10 and 36/cm^2^. The follicle density in the Kasting et al. 2019 [12] model is scaled down by a dimensionless parameter $f_{open}$, which represents the proportion of open follicles. This quantity has a nominal value of 0.015 in reference [12]. For the purposes of sensitivity analysis, $N_{f}$ and $f_{open}$ can be viewed as a single lumped parameter since they enter the model together, as a product.
- The follicle orifice radius, $r_{1}$, ranges between 4-fold and 6-fold the radius of the hair shaft ($r_{0}$) as per Otberg et al. [16], whereas the nominal value recommended in [13] yields $r_{1}/r_{0}$ = 1.25. Given the large uncertainty, the range was taken to be 1–10.
- The transcellular pathway parameters $r_{2}$ and $N_{p}$ are novel quantities that were introduced in [12]. As such, the literature does not provide reliable uncertainty estimates for these parameters. For this reason, they are assumed to vary within an order of magnitude of their nominal proposed values in reference [12].

**Table 5:** Fixed values for skin-specific parameters of the skin permeation model. See Nomenclature.

Parameter	Description	Value	Reference
$h_{ed}$	Viable epidermis thickness	100 µm	[29]
$h_{de}$	Dermis thickness for heat-separated epidermis skin	0 µm	[22]
	Dermis thickness for dermatomed skin	100 µm	

**Table 6:** Uncertainties in skin-specific parameters of the skin permeation model in Appendix A. See Nomenclature.

Parameter	Description	Value	Units	Reference
*Stratum corneum parameters*				
$h_{SC}$	Stratum corneum thickness (fully hydrated)	Nominal: 29 (thigh), 43.4 (abdomen) Range: 13–65 (thigh), 19–97 (abdomen)	µm	Table 6 in [27]
$m_{y}$	Lipid bilayer envelope + corneocyte thickness (fully hydrated SC)	Nominal: 2.9 Range: 2.32–3.63	µm	Nominal: [19] Range: [28]
*Follicle pathway parameters*				
$r_{1}/r_{0}$	Ratio of follicle orifice radius to hair shaft radius	Nominal: 4.59 (thigh), 5.74 (abdomen) Range: 1–10		[16]
$N_{f}$	Number of follicles per area	Nominal: 18 (thigh), 21 (abdomen) Range: 12–36	cm^−2^	[16]
$f_{open}$	Proportion of open follicles	Nominal: 0.015 Range: Not reported		Nominal: [13]
*Transcellular porous pathway parameters*				
$r_{2}$	Micropore radius	Nominal: 1.6 Range: 0.16–16	nm	Nominal: [12] Range: Assumed
$N_{p}$	Number of micropores per area	Nominal: 373,000 Range: 37,300–3,730,000	cm^−2^	Nominal: [12]

## 11. Sensitivity Analysis

**Figure 5:**
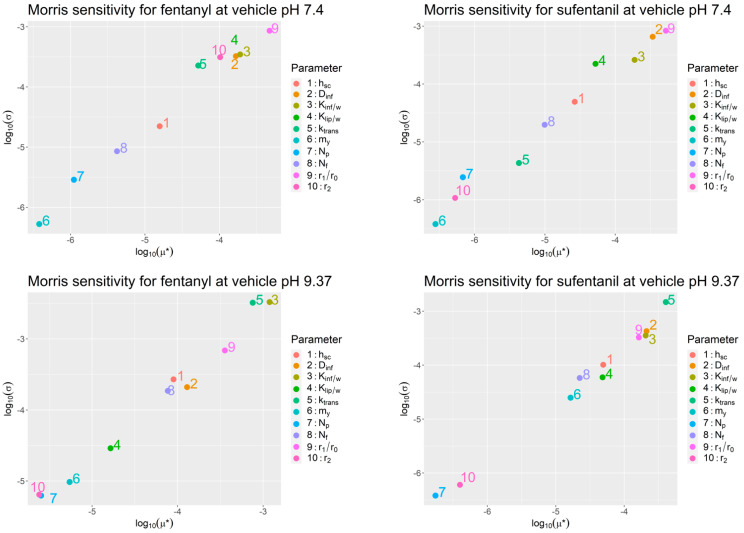
Morris sensitivity analysis results for fentanyl and sufentanil at vehicle pH levels 7.4 and 9.37.

Morris sensitivity screenings were conducted for fentanyl and sufentanil at vehicle pH levels of 7.4 and 9.37 (Figure 5). Both compounds are bases that are significantly ionized at pH 7.4 and largely non-ionized at pH 9.37. For this reason, the permeability across the non-polar pathway, $k_{trans}$, has a much larger influence on aggregate permeability $P_{tot/w}$ at pH 9.37 than it does at pH 7.4. On the other hand, the micropore density and micropore radius $r_{2}$, which are descriptors of the polar pathway across the stratum corneum, have a more influential role at pH 7.4 as they would more strongly contribute to the permeability by admitting ionized solutes. However, highly influential parameters at both pH levels were descriptors of the infundibular pathway, which provides a permeation pathway that bypasses the stratum corneum entirely. Of all the parameters, the micropore pathway parameters $r_{2}$ and $N_{p}$ showed low influence on $P_{tot/w}$ for both compounds and both vehicle pH levels. As a result, this parameter was not included among the parameters to be inferred from experimental data.

## 12. Prior Distributions of Model Parameters to Be Inferred

Based on the sensitivity and uncertainty analysis, prior distributions were defined for the parameters to be inferred from the experimental data in Groups A–E, and these are summarized in Table 7. All parameters to be inferred were defined as dimensionless scalings or additive perturbations to the nominal values of their “parent” parameters in Table 4 and Table 6. Parameters that are liable to vary between individuals and/or across anatomical sites were defined as being either individual-specific, site-specific, or both.

Table 6 in [27] summarizes literature-sourced measurements of the adult stratum corneum thickness $h_{SC}$ from multiple body regions. These measurements indicate a high degree of variability in $h_{SC}$ between different body regions. For this reason, we decomposed $h_{SC}$ as $h_{SC}=h_{SC}^{nom}\times h_{SC}^{site}\times h_{SC}^{ind}$, where $h_{SC}^{nom}$ is the proposed nominal value for hydrated skin in [10] and the parameters $h_{SC}^{site}$ and $h_{SC}^{ind}$ are dimensionless scaling factors that quantify the intra-region variability and intra-individual variability, respectively, in stratum corneum thickness. The nonparametric joint posterior distribution inferred by the MCMC algorithm therefore includes two random variables $h_{SC}^{site}$, one for each of the abdomen and the thigh regions. In addition, the joint distribution includes a random variable $h_{SC}^{ind}$ for each individual. The distributions of the scalings $h_{SC}^{site}$ thus quantify the deviations of the SC thickness from the nominal value $h_{SC}^{nom}$ for each body region, while the scalings $h_{SC}^{ind}$ will quantify the different individuals’ deviations in SC thickness from the product $h_{SC}^{nom}\times h_{SC}^{site}$. Based on the sensitivity analysis, the total thickness of the stratum corneum lipid bilayer envelope and the total corneocyte thickness $m_{y}$ were shown to have little effect on permeability, and were therefore not selected for inference.

The permeability across lipid bilayers $\log_{10}k_{trans}$ was assumed to have both a compound-specific and individual-specific effect. The compound plays a role due to the correlation with molecular weight highlighted in Wang et al. [19]. We assume an individual-level effect to account for variability in the structure of stratum corneum lipids between individuals.

The influential partition coefficients $K_{lip/w}$ and $K_{inf/w}$ were only assumed to have a compound-specific effect that scales them with respect to their nominal values, which depend on $\log_{10}K_{o/w}$ and $pK_{a}$, respectively. On the other hand, the diffusivity within the infundibulum was assumed to have an individual-specific effect.

Otberg et al. [16] reported that both the ratio of infundibular radii $\left(r_{1}/r_{0}\right)$ and the follicle density $N_{f}$ vary across anatomical sites. Within the model, these two quantities modify the infundibular cross-sectional area. For this reason, we lump their effect into $N_{f}$ and regard it as a parameter that varies between anatomical sites. On the other hand, we regard the proportion of open follicles $f_{open}$ to be an individual-specific parameter. Finally, in agreement with the observations in [13], the sensitivity analysis showed that the micropore route across stratum corneum lipids has little impact on permeability compared to the follicular route. The micropore parameters $r_{2}$ and $N_{p}$ were therefore not included among the quantities to be inferred.

## 13. Internal Validation

**Figure 6:**
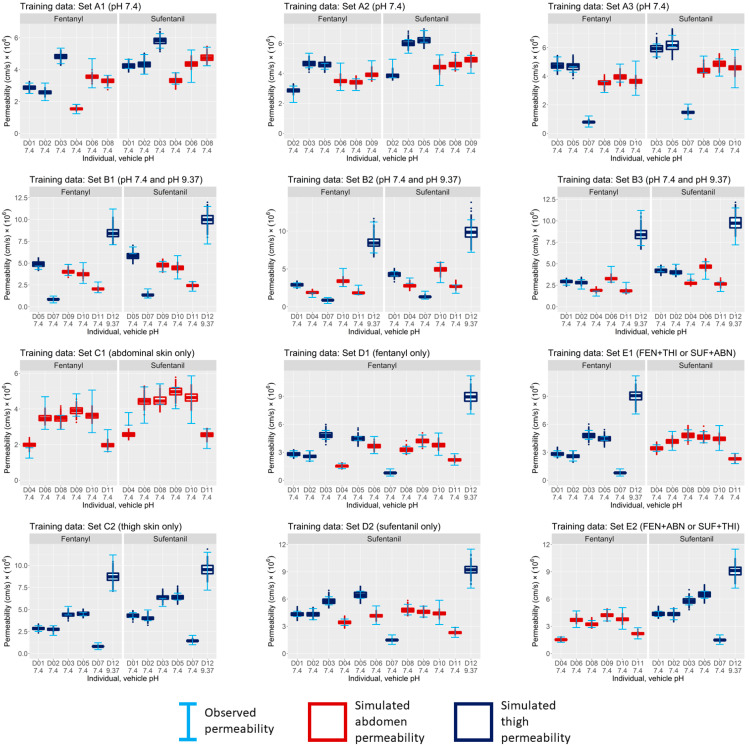
Visual predictive check for internal validation of observed vs. simulated permeability for data sets in Groups A, B, E. Error bars represent the measured permeability range for each individual in the training data set. The box plots in each panel represent the distributions of the estimated permeability for each individual after training the model using the respective data set. FEN = fentanyl, SUF = sufentanil, ABN = abdominal skin, THI = thigh skin.

Following the application of the MCMC algorithm, the goodness of fit of the model’s permeability estimates to experimental data was evaluated based on the learned posterior distribution. This evaluation was completed for each individual within each training data set by repeatedly simulating the model using compound-specific, site-specific, and individual-specific parameter samples from the joint posterior distribution inferred from the permeability measurements. The results of the simulations provide the range of estimations of fentanyl and sufentanil permeability based on the learned distributions. These permeability ranges are shown in Figure 6 for Groups A–E. For comparison, these estimates are shown alongside their corresponding experimental measurements. The figure demonstrates that, for all data sets, the learned joint posterior distributions yield permeability estimates that agree well with the experimentally observed permeability ranges.

## 14. External Validation

We next validate the inferred posterior distributions by comparing model estimates of permeability with measurements that are outside the scope of the training data.

### 14.1. External Validation 1

**Figure 7:**
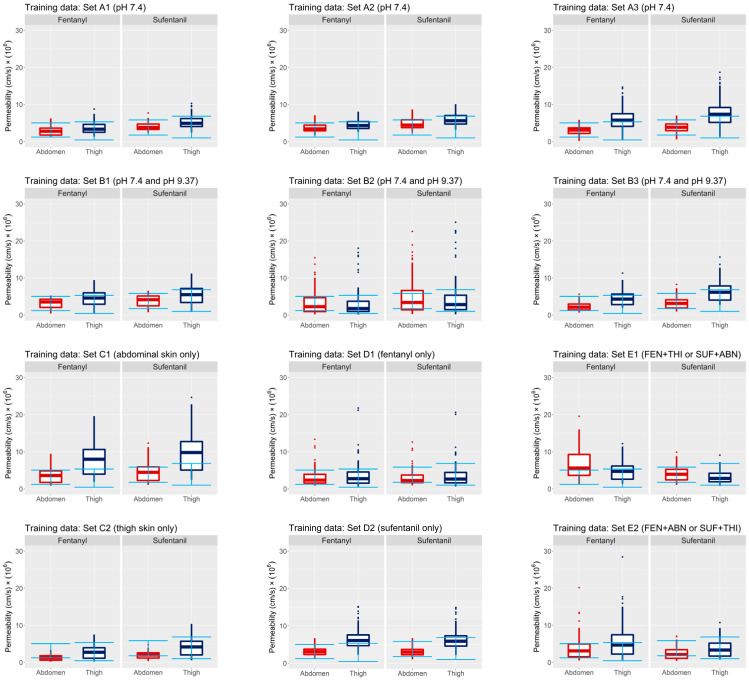

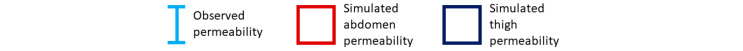
External validation of trained model against permeability measurements at vehicle pH 7.4. Error bars represent the range of observed permeabilities for each compound and anatomical site for all individuals in Table 1. Each box plot shows the distributions of estimated permeability based on individual-specific parameters inferred from the respective data set. FEN = fentanyl, SUF = sufentanil, ABN = abdominal skin, THI = thigh skin.

The first external validation is performed using the permeability measurements in Table 1, which summarizes the results of experiments conducted at vehicle pH 7.4. The error bars in Figure 7 reflect the range of permeability values observed across all individuals for a given compound and anatomical site in Table 1. Each set of box plots in Figure 7 shows the distribution of the permeability estimated in model simulations. The estimates in each panel are generated by simulating the model using samples from the joint posterior distributions inferred from their respective data sets. These estimates are shown for fentanyl and sufentanil, applied to abdominal and thigh skin, with individual-specific parameters sampled from those inferred for the group of donors in each data set.

Good agreement between the extrapolated model estimates and observations can be seen in the cross-over design Groups A and B, with all the median permeability estimates falling within the observed range for Group B.

The external validation of Group C data sets highlights that extrapolating across anatomical sites can lead to biased estimates of permeability. When the model was trained with data set C1, comprising exclusively abdominal skin samples, it exhibited an overprediction of fentanyl and sufentanil permeability in thigh skin, while accurately predicting permeability in abdominal skin. Conversely, when the model was trained using permeability data solely from thigh skin samples, it resulted in an underestimation of permeability in abdominal skin but demonstrated accurate predictions for thigh skin measurements.

Validation of the Group D data sets generally showed concordance between the simulated and observed permeability. The model trained with data set D1 generated permeability estimates in agreement with the observed sufentanil permeability across both abdominal and thigh skin. Additionally, the model trained with sufentanil permeability data (data set D2) demonstrated accurate predictions for fentanyl permeability across abdominal skin, although it slightly overestimated fentanyl permeability in thigh skin.

Group E simulations generally showed good concordance with observed permeability estimates. However, there was some mild bias in the estimates of the model when extrapolated to predict abdominal permeability of fentanyl by the model when trained with fentanyl applied to thigh skin and sufentanil applied to abdominal skin (data set E1). In all other Group E cases, the median estimate fell within the observed range.

### 14.2. External Validation 2

**Figure 8:**
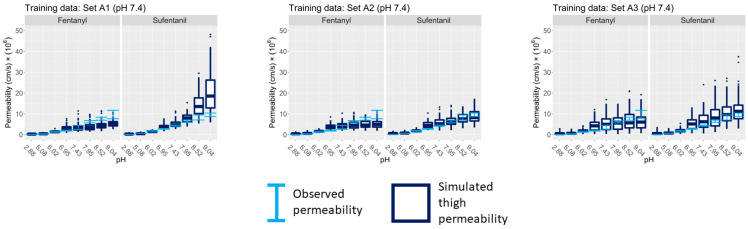
External validation of the trained model against permeability measurements at vehicle pH 2.88–9.04. Error bars represent permeability measurements for individual D12 for all vehicle pH levels (from Table 2). The box plots represent the estimated permeability given the variability inferred from the entire donor population in the training data sets A1 (**left**), A2 (**middle**), and A3 (**right**).

**Figure 9:**
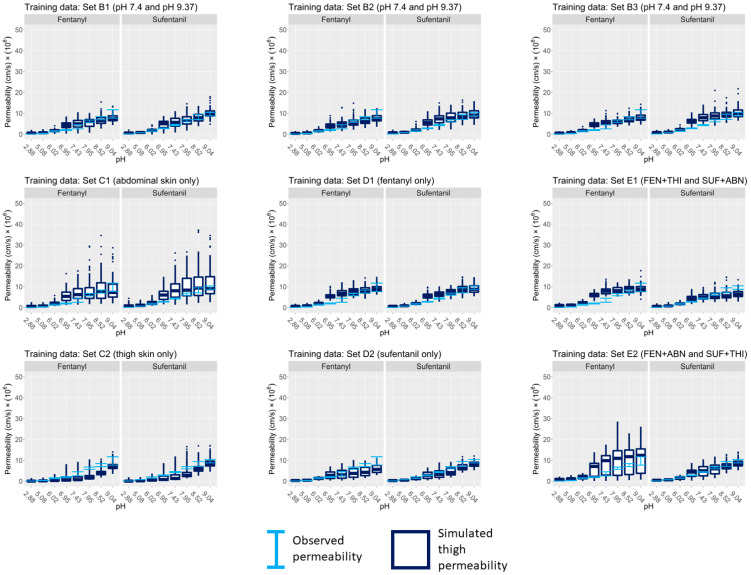
External validation of trained model against permeability measurements at vehicle pH 2.88–9.04 for individual D12. Error bars represent permeability measurements taken from a single individual for all vehicle pH levels. These measurements are given in Table 2. The box plots represent the estimated permeability given the variability inferred from the entire population in the training data set. FEN = fentanyl, SUF = sufentanil, ABN = abdominal skin, THI = thigh skin.

The second external validation compares the model-generated permeability estimates against the experiments in Table 2 in which the vehicle pH was between 2.88 and 9.04. The results are shown in Figure 8 for Group A data sets and Figure 9 for data sets in Groups B–E. Error bars in both figures, across all vehicle pH levels, represent measurements of permeability across thigh skin sections taken from individual D12.

Since Group A data sets did not include individual D12, the box plots in each panel in Figure 8 represent the range of permeability estimates for all individuals within each respective Group A data set. On the other hand, the box plots in Figure 9 show distributions of estimated permeability that are generated by the dermal model using inferred parameter samples specific to individual D12. For this reason, the permeability estimates for Group A show generally wider confidence intervals than for Groups B–E.

When extending the model initially trained with abdominal skin permeability measurements to predict permeability across thigh skin, wide confidence intervals in the estimated permeability were obtained, as in the cases of data sets C1 and E2.

## 15. Analysis of Pathway-Specific Permeability

**Figure 10:**
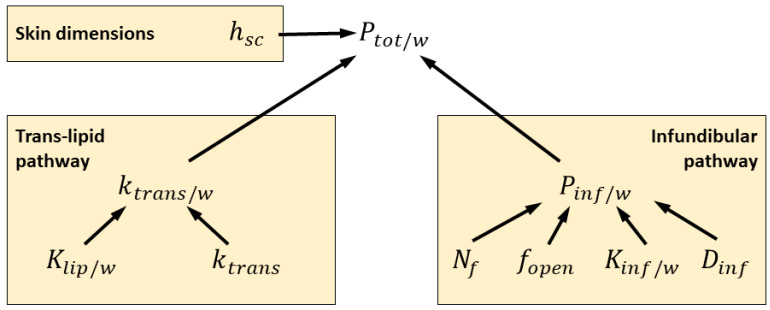
Functional dependence of the aggregate permeability $P_{tot/w}$ on pathway-specific permeabilities $k_{trans/w}$ and $P_{inf/w}$ and their underlying parameters inferred in this study.

Figure 10 shows the hierarchical dependence of the total permeability $P_{tot/w}$ on the trans-stratum corneum lipid bilayer pathway and the infundibular pathway. The figure also shows the dependence of these pathway-specific permeabilities on underlying model parameters that were inferred from data in this study.

The sensitivity analysis results in Figure 5 show that the parameters underlying the infundibular pathway permeability $P_{inf/w}$ most influence the aggregate permeability $P_{tot/w}$ when the vehicle pH is 7.4. At this pH, fentanyl and sufentanil are strongly ionized. For this reason, the infundibulum pathway constitutes a pathway for polar solutes.

The sensitivity analyses also show that the permeability across lipid bilayers, $k_{trans}$, is highly influential with respect to both fentanyl and sufentanil permeability at high pH, when the two compounds are highly non-ionized. The same figure shows that this parameter plays a significantly less dominant role at near-neutral pH levels.

Partitioning of the permeant into stratum corneum lipids is denoted $K_{lip/w}$. The product of this quantity and $k_{trans}$ gives the trans-lipid bilayer permeability relative to water, which we denote by $k_{trans/w}$. This product is of significance in the following analysis because, as discussed in Appendix A, it enters directly into the calculation of permeability of the non-polar pathway $P_{tot}^{non}$.

We have assumed that $K_{lip/w}$ is a compound-specific property and that $k_{trans}$ includes both a compound-specific and an individual-specific effect, the latter of which is to account for potential individual-level variability in the stratum corneum lipid matrix structure. As detailed in Table 7, compound-specific scalings of $K_{lip/w}$ and $k_{trans}$ and individual-specific scalings of $k_{trans}$ were inferred from the experimental data.

**Figure 11:**
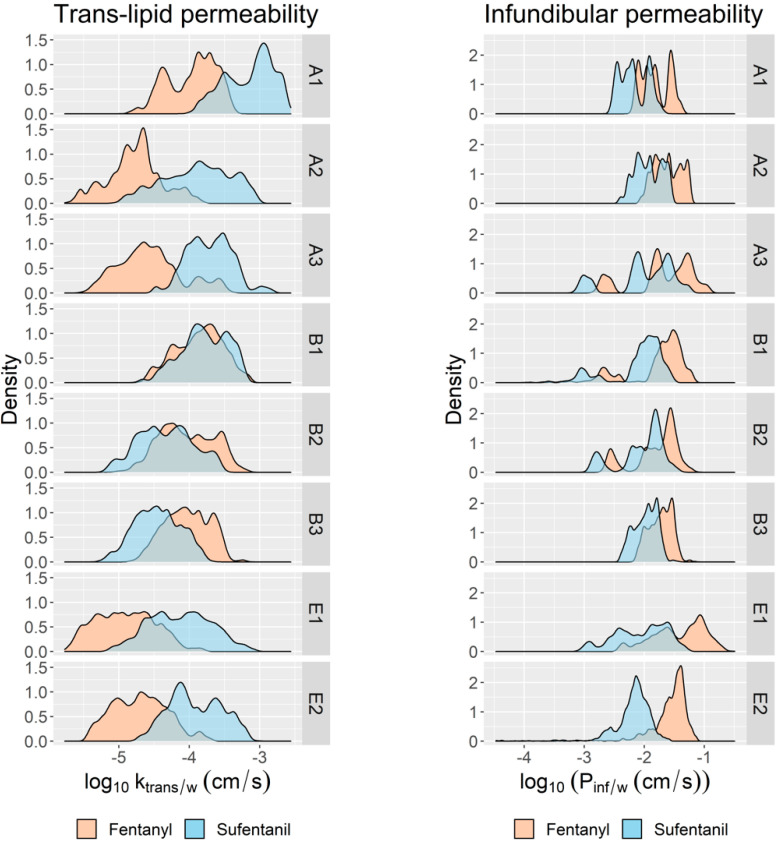
Marginal distributions of pathway-specific permeabilities for fentanyl and sufentanil inferred from data sets in Groups A, B, and E. (**Left**) The trans-lipid bilayer permeability relative to water $\left(\log_{10}k_{trans/w}\right)$, the most influential parameter in the non-polar pathway. (**Right**) Infundibular pathway permeability.

By sampling these scalings from the inferred posterior distributions and evaluating the product of each set of samples with the nominal values of $K_{lip/w}^{nom}$ and $k_{trans}^{nom}$, we generated distributions of $k_{trans/w}$, as shown in Figure 11 (left panel), for both compounds and each data set in Groups A, B, and E. The commonality between these groups is that their data sets included experiments conducted using both fentanyl and sufentanil and skin samples from both the thigh and abdomen. In a similar way, we constructed posterior distributions of the infundibular pathway permeability $P_{inf/w}$ by sampling its underlying parameters in Figure 10 from the inferred joint distributions. These distributions are shown in Figure 11 (right panel).

## 16. Discussion

The promise of model-informed decision making in formulation design and risk assessment is the capability to safely bypass resource-intensive experimentation and clinical trialing through a leveraging of prior observations and knowledge of the mechanisms of dermal disposition. However, this process relies on confidence in modeled predictions. The credibility of pharmacokinetic models is strengthened by “stressing” them to simultaneously mechanistically explain diverse data sets that cut across different drugs, populations, and application scenarios. In this way, important and reliable estimates of the range, variability, and correlations between pharmacokinetic quantities can be learned for use in future predictions. Thus, it has been the aim of this work to examine how the choice of data sets used in model training impacts the ability of models to learn inter-site and inter-individual variability and to generate accurate and precise yet non-conservative extrapolations based on these inferences.

Central to this workflow is the dermal model. We have further developed the MoBi mechanistic dermal model [11] to incorporate the follicular and trans-cellular polar pathways proposed in Kasting et al. [12] and Yu et al. [13]. With this update, we adapted the MoBi model to simulate permeability across different body regions by adjusting site-specific parameters such as follicle density (see Table 6). In addition, this update enables the adjustment of permeability to account for the pH-dependent ionization state of the solute in the vehicle, as described in Appendix A. Both fentanyl and sufentanil are lipophilic (Table 3) and basic compounds with respective pK_a_ values of 8.99 and 8.56, and are, therefore, partially ionized in the pH 7.4 vehicles used in the experiments summarized in Table 1 and largely non-ionized in the high pH experiments in Table 2. This work therefore provides a case study in the training and application of the dermal model proposed by Yu et al. [13] to estimate skin permeation by compounds that may be transported via both polar and non-polar pathways. This is especially important in contexts where a topically applied formulation may undergo metamorphosis due to, for example, the evaporation of components such as water. In such cases, the ionization state, and thus the skin permeability, of active pharmaceutical ingredients may change over time.

## 17. Model Extrapolation across Anatomical Sites and Compounds

The outputs of the learning algorithm employed in this study are encompassed in nonparametric probability distributions that summarize the likely ranges and correlations of quantities that scale compound-, site-, and individual-specific model parameters. The extrapolations of model parameters inferred from the Group C and D data sets, shown in Figure 7 and Figure 9, provide an assessment of the feasibility and limitations of this approach. The parameters learned from Group C data sets were extrapolated to contexts involving skin from anatomical sites that differ from those used in the training data. Data set C1 only included abdominal skin samples. Figure 7, panel C1, shows that when the model that was trained on abdominal skin data was subsequently extrapolated to simulate permeability in thigh skin, there was an overestimation of the range of permeability of both fentanyl and sufentanil. The opposite effect is observed when the model trained on thigh skin is extrapolated to estimate abdominal skin permeability—here, the permeability range is underestimated for both compounds.

Panels D1 and D2 in Figure 7 and Figure 9 show that the extrapolation of inferred parameters across compounds yielded good fits to permeability measurements. This demonstrates that samples of compound-specific scalings $\theta_{c}$ can be drawn from their inferred joint posterior distribution and used in extrapolating the model from one compound to another. Since they are drawn from a joint distribution, these scalings contribute informational value to the extrapolation because they maintain the correlations that were identified during the training phase. These findings underscore the feasibility of learning skin-specific properties in one population using a single compound and subsequently applying this knowledge to predict permeability for other compounds within the same population.

## 18. Effect of Experiment Design on Parameter Calibration

Given the potential value of the inferred posterior distributions in risk assessment and drug design, an examination of the robustness of these quantities is merited. The posterior distributions corresponding to each data set enable us to examine how the various experimental designs impact the inferred distributions of the mechanistic parameters of the model.

### 18.1. Inclusion of High pH Vehicle Experiments Improves Calibration of Non-Polar Pathway Permeability

Figure 11 (left panel) shows that the fentanyl and sufentanil estimates of $k_{trans/w}$ diverge in all three Group A data sets. This is contrary to what might be expected since fentanyl and sufentanil have similar molecular weights, lipophilicities, and pKa values and should therefore have similar permeabilities across a given medium. Here, it should be noted that Group A only included experiments in which the vehicle pH level was 7.4, thus blinding the model, during the training phase, to measurements in which the fentanyl or sufentanil was non-ionized. Essentially, this divergence indicates that $k_{trans/w}$ is mis-calibrated when the data sets only include experiments involving pH 7.4 since that parameter does not strongly influence permeability when the solute is ionized. The divergence also explains why, when extrapolating the model trained using pH 7.4 experiments to high pH contexts, where $k_{trans/w}$ is influential, we observe scenarios in which there is an underestimation of fentanyl permeability and a simultaneous overestimation of sufentanil permeability, as seen for set A1 in Figure 8. In contrast, Group B, which included a pH 9.37 experiment in each data set, shows much closer agreement in $k_{trans/w}$ between the two compounds.

### 18.2. “Cross-Over” Design Mitigates Correlations between Polar and Non-Polar Pathway Permeabilities

Figure 11 shows that the Group E estimates of both non-polar (left panel) and infundibular (right panel) pathways show strong divergence between the fentanyl and sufentanil estimates. Moreover, the Group E estimates suggest that fentanyl simultaneously has a significantly larger infundibular permeability, and a significantly lower non-polar pathway permeability, than sufentanil. As before, it is unlikely that such large differences exist between the two compounds. Although the Group B estimates also show divergence in permeabilities between the two compounds, it is to a relatively small extent.

Both the Group B and Group E training data sets include fentanyl and sufentanil experiments, with vehicles at both pH 7.4 and pH 9.37, applied to both abdominal and thigh skin, from multiple individuals. The difference between the two data sets lies in the “cross-over” versus “parallel” designs of the training data sets in Groups B and E, respectively. In Group B, both compounds are applied to skin from each individual within the data set population, whereas in Group E, each compound is applied to skin from a different set of individuals.

Permeabilities $k_{trans/w}$ and $P_{inf/w}$ are functions of individual-specific as well as compound-specific quantities and together contribute to the aggregate permeability $P_{tot/w}$ for each compound. They are therefore correlated quantities in the model. The cross-over design of Group B trains the model to estimate the aggregate permeability $P_{tot/w}$ for the two compounds across skin sourced from the same individual. This ensures that the individual-specific contribution to permeabilities $k_{trans/w}$ and $P_{inf/w}$ for each compound is the same. In this way, the Group B estimates exclude parameter combinations from their posterior distributions in which $k_{trans/w}$ is overestimated and $P_{inf/w}$ is underestimated for one compound but not the other. By excluding such parameter combinations from the inferred distributions, Group B estimates avoid the divergences between fentanyl and sufentanil estimates of $k_{trans/w}$ and $P_{inf/w}$ that are seen with Group E.

## 19. Limitations

### 19.1. A Need for More Diverse Compounds for Model Evaluation

The external validation of the model trained under sets D1 and D2 tested the ability of the proposed workflow to learn the distributions of individual-specific and site-specific model parameters from experiments conducted using one compound, and to then leverage this information to predict the skin permeability of another compound. However, this validation test has limitations because the two compounds used in assessing the proposed workflow, fentanyl and sufentanil, are similar in molecular weight, lipophilicity, and ionizability. Consequently, the extrapolation from one compound to another is expected to yield good fits to the validation data. Therefore, further evaluation of the workflow using a variety of compounds with a diversity of molecular weights, lipophilicities, and degrees of ionizability is needed.

### 19.2. Knowledge of Parameter Priors Is Limited

As the model used in this work is relatively recent, the variability in some of the quantities that determine dermal permeability within the model has yet to be experimentally measured. These include the parameters in Table 7, several of which have highly uncertain prior distributions. This uncertainty can bias the inferred posterior distributions or otherwise limit them to incorrect ranges. Furthermore, prior knowledge of the model parameters impacts the sensitivity analyses. Therefore, a mischaracterization of the prior distributions can lead to the omission of important parameters from the model training. However, the posterior distributions that were inferred for these parameters in this study can serve as prior distributions in future work, thereby enhancing the predictive performance of the model.

## 20. Future Applications to Chemical Risk Assessment

In future work, the methodology adopted in this study can also be used to learn the distributions of skin descriptors for anatomical sites beyond the abdomen and thigh regions. In addition, this approach can be used to learn the distribution of skin-specific parameters in special populations such as the elderly or individuals with diseased or compromised skin.

## 21. Conclusions

In this study, a learning and extrapolation workflow was employed to train and evaluate a mechanistic dermal model using skin permeability measurements. The choice of measurements utilized for model training was identified as a critical factor influencing the model’s accuracy and precision in predicting skin permeability under novel contexts. Notably, incorporating a diverse range of experimental scenarios and implementing a cross-over study design within the training data set significantly enhanced the model’s overall performance in these aspects.

## Figures and Tables

**Table 1:** Experimentally measured permeabilities of fentanyl and sufentanil at pH 7.4 across heat-separated epidermis, as reported in reference [22].

Individual	pH	Region	Number of Replicates	Fentanyl		Sufentanil	
				Experiment Name	Permeability $P_{tot/w}$ (×10^6^) cm/s Mean (SD)	Experiment Name	Permeability $P_{tot/w}$ (×10^6^) cm/s Mean (SD)
D01	7.40	Thigh	4	F01	2.83 (0.28)	S01	4.36 (0.25)
D02	7.40	Thigh	4	F02	2.61 (0.47)	S02	4.33 (0.53)
D03	7.40	Thigh	4	F03	4.86 (0.42)	S03	5.81 (0.39)
D04	7.40	Abdomen	4	F04	1.53 (0.25)	S04	3.44 (0.31)
D05	7.40	Thigh	4	F05	4.47 (0.17)	S05	6.47 (0.33)
D06	7.40	Abdomen	5	F06	3.78 (0.72)	S06	4.22 (0.81)
D07	7.40	Thigh	5	F07	0.83 (0.31)	S07	1.53 (0.42)
D08	7.40	Abdomen	4	F08	3.25 (0.33)	S08	4.83 (0.50)
D09	7.40	Abdomen	5	F09	4.22 (0.50)	S09	4.61 (0.47)
D10	7.40	Abdomen	5	F10	3.86 (0.94)	S10	4.53 (1.06)
D11	7.40	Abdomen	4	F11	2.22 (0.53)	S11	2.33 (0.47)

**Table 2:** Summary of fentanyl and sufentanil permeabilities across thigh dermatomed skin sections at various vehicle pH levels, as reported in Roy and Flynn, 1990 [22].

Individual	pH	Region	Number of Replicates	Fentanyl		Sufentanil	
				Experiment Name	Permeability $P_{tot/w}$ (×10^6^) cm/s Mean (SD)	Experiment Name	Permeability $P_{tot/w}$ (×10^6^) cm/s Mean (SD)
D12	2.88	Thigh	4	FpH1	0.08 (0.01)	SpH1	0.13 (0.01)
D12	5.08	Thigh	4	FpH2	0.36 (0.08)	SpH2	0.69 (0.03)
D12	6.02	Thigh	4	FpH3	1.42 (0.22)	SpH3	1.72 (0.31)
D12	6.95	Thigh	4	FpH4	1.97 (0.19)	SpH4	2.81 (0.17)
D12	7.43	Thigh	4	FpH5	3.53 (0.83)	SpH5	4.36 (0.22)
D12	7.95	Thigh	4	FpH6	6.22 (0.47)	SpH6	6.42 (0.44)
D12	8.52	Thigh	4	FpH7	7.67 (0.64)	SpH7	8.28 (1.03)
D12	9.04	Thigh	4	FpH8	9.69 (1.75)	SpH8	9.58 (0.69)
D12	9.37	Thigh	4	FpH9	9.14 (1.75)	SpH9	9.36 (1.86)

**Table 7:** Prior distributions of model parameters to be inferred.

Parent Parameter Decomposition	Inference Parameter Prior Distributions
$h_{SC}=h_{SC}^{nom}\times h_{SC}^{site}\times h_{SC}^{ind}$ Nominal value: $h_{SC}^{nom}$ from Table 6. Site-specific scaling: $h_{SC}^{site}$ Individual-specific scaling: $h_{SC}^{ind}$	$h_{SC}^{site}$~LogUniform(1/1.5, 1.5) $h_{SC}^{ind}$~LogUniform(1/1.5, 1.5)
$\log_{10}k_{trans}$ (cm/s) = $\log_{10}k_{trans}^{nom}$ + $\log_{10}k_{trans}^{cmp}$ + $\log_{10}k_{trans}^{ind}$ Nominal value: $\log_{10}k_{trans}^{nom}$ from Table 4. Compound-specific additive perturbation: $\log_{10}k_{trans}^{cmp}$ Individual-specific additive perturbation: $\log_{10}k_{trans}^{ind}$	$\log_{10}k_{trans}^{cmp}$~Uniform(−0.54, 0.54) $\log_{10}k_{trans}^{ind}$~Uniform(−0.54, 0.54)
$\log_{10}K_{lip/w}$ = $\log_{10}K_{lip/w}^{nom}$ $+\log_{10}K_{lip/w}^{cmp}$ Nominal value: $\log_{10}K_{lip/w}^{nom}$ from Table 4. Compound-specific additive perturbation: $\log_{10}K_{lip/w}^{cmp}$	$\log_{10}K_{lip/w}^{cmp}$~Uniform(−0.43, 0.43)
$\log_{10}K_{inf/w}$ = $\log_{10}K_{inf/w}^{nom}$ + $\log_{10}K_{inf/w}^{cmp}$ Nominal value: $\log_{10}K_{inf/w}^{nom}$ from Table 4. Compound-specific additive perturbation: $\log_{10}K_{inf/w}^{cmp}$	$\log_{10}K_{inf/w}^{cmp}$~Uniform(−1, 1)
$\log_{10}D_{inf}$ (cm^2^/s) = $\log_{10}D_{inf}^{nom}$ + $\log_{10}D_{inf}^{ind}$ Nominal value: $\log_{10}D_{inf}^{nom}$ from Table 6. Individual-specific additive perturbation: $\log_{10}D_{inf}^{ind}$	$\log_{10}D_{inf}^{ind}$~Uniform(−1, 1)
$\log_{10}f_{open}$ = $\log_{10}f_{open}^{nom}$ + $\log_{10}f_{open}^{ind}$ Nominal value: $\log_{10}f_{open}^{nom}$ from Table 6. Individual-specific additive perturbation: $\log_{10}f_{open}^{ind}$	$\log_{10}f_{open}^{ind}$~Uniform(−1, 1)
$N_{f}$ = $N_{f}^{nom}$ × $N_{f}^{site}$ Nominal value: $N_{f}^{nom}$ from Table 6. Site-specific scaling: $N_{f}^{site}$	$N_{f}^{site}$~LogUniform(1/1.25,1.25)

**Figure A1:**
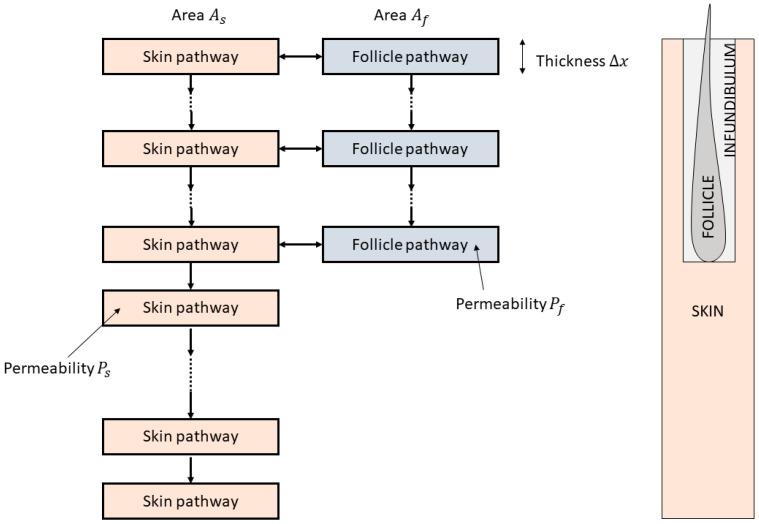
Schematic illustration of the MoBi implementation of the follicular pathway.

**Figure A2:**
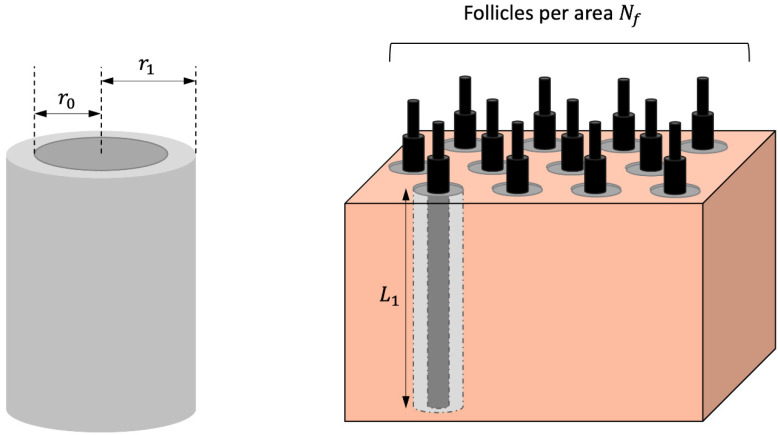
Follicle pathway dimensions.

**Figure A3:**
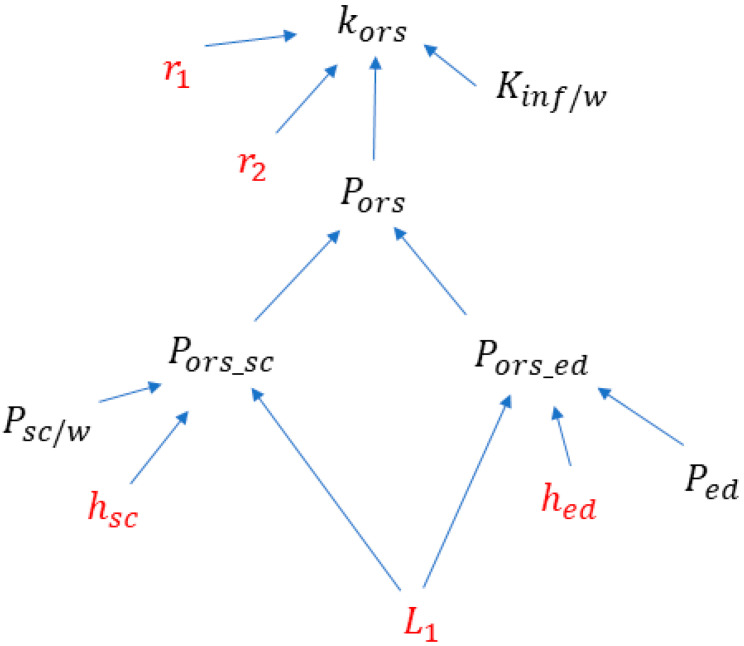
Decomposition of the skin–infundibulum mass transfer coefficient $k_{ors}$.

**Figure A4:**
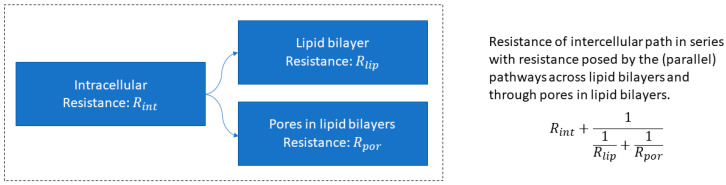
Parallel transcellular pathway through the SC via pores in the SC lipids.

## Data Availability

All data used in this study are included within the manuscript.

## Nomenclature

$K_{sc/w}$	Partition coefficient of permeant in stratum corneum relative to water.
$P_{sc/w}$	Permeability of permeant in stratum corneum.
$D_{sc}$	Stratum corneum diffusion coefficient.
$K_{ed/w}$	Partition coefficient of permeant in viable epidermis relative to water.
$P_{ed}$	Permeability of permeant in epidermis.
$D_{ed}$	Viable epidermis diffusion coefficient.
$K_{de/w}$	Partition coefficient of permeant in dermis relative to water.
$D_{de}$	Dermis diffusion coefficient.
$K_{inf/w}$	Partition coefficient of permeant in infundibulum relative to water.
$D_{inf}$	Infundibulum diffusion coefficient.
$K_{o/w}$	Partition coefficient of permeant in octanol with respect to water.
$K_{lip/w}$	Partition coefficient of permeant in SC lipids with respect to water.
$K_{cor/w}$	Partition coefficient of permeant in SC corneocyte phase with respect to water.
$D_{cor}$	Diffusion coefficient of permeant in SC corneocyte phase.
$PC_{pro/w}$	Partition coefficient of permeant in corneocyte protein phase with respect to water.
$P_{ors-sc}$	Permeability of permeant in SC-like tissue surrounding follicle.
$P_{ors-ed}$	Permeability of permeant in epidermis-like tissue surrounding follicle.
$P_{ors}$	Aggregate permeability of permeant in tissue surrounding follicle.
$k_{trans}$	Trans-lipid-bilayer permeability in stratum corneum.
$r_{0}$	The radius of the follicle shaft.
$r_{1}$	The radius of the follicle shaft.
$L_{1}$	The length of the follicle.
$N_{f}$	The number of follicles per unit area of skin surface.
$f_{open}$	Proportion of open follicles
$h_{sc}$	Stratum corneum thickness.
$h_{ed}$	Epidermis thickness.
$h_{de}$	Dermis thickness.
$m_{y}$	Lipid bilayer envelope + corneocyte thickness
$\lambda_{3}$	Ratio of the permeant radius to the micropore radius.
$f_{non/veh}$	Fraction of permeant amount in the vehicle that is non-ionized.
$f_{non/veh}$	Fraction of permeant amount in water that is non-ionized.
$f_{non/sc}$	Fraction of permeant amount in the stratum corneum that is non-ionized.

## Appendix A. Mathematical Model

We have previously implemented the Dancik et al. [10] skin permeation model in MoBi [11]. This model regards the dermal application area to be a uniform, three-layered slab composed of the stratum corneum, the viable epidermis, and the dermis. The three layers have respective thicknesses $h_{sc}$, $h_{ed}$, $h_{de}$. Partition coefficients of a given compound with respect to the compound’s non-ionized form in water are denoted as $K_{sc/w}$, $K_{ed/w}$, $K_{de/w}$, and the diffusion coefficients corresponding to the respective layers are denoted as $D_{sc}$, $D_{ed}$, $D_{de}$.

For most compounds, the stratum corneum provides the most significant barrier of the three layers. For this reason, the stratum corneum diffusivity, $D_{sc}$, is an important metric of a compound’s permeability in this model [21]. A mechanistic decomposition of this diffusivity was proposed in [19] and adopted into the model of Dancik et al. [10]. As shown in [21], $D_{sc}$ is strongly influenced by the active compound’s permeability ($k_{trans}$) across the lipid bilayers that envelope the corneocytes of the stratum corneum. A further component of $D_{sc}$ is the permeant’s diffusivity through corneocytes, $D_{cor}$.

An additional parameter of importance is the partition coefficient into the stratum corneum, $K_{sc/w}$. In [26], this aggregate quantity was decomposed mechanistically and expressed as a function of the active compound’s partitioning into the SC lipids ($K_{lip/w})$ and into the SC corneocytes ($K_{cor/w}$).

### Appendix A.1. Pathway A—Follicular pathway of Yu et al., 2021 [13]

This modification provides a follicular route through which hydrophilic solutes may bypass the stratum corneum directly into viable epidermis and dermis. A schematic representation of the MoBi implementation of this pathway is shown in Figure A1. The parallel, ladder-like structures represent segments of the skin and follicular pathways through which the permeant may be transported. As shown in this figure, the permeant may diffuse both downwards, deeper into the skin, and laterally, between the skin and the infundibular region of the follicular pathway.

This pathway is parameterized by the following:

- the dimensions and density of the follicles and the infundibulum (Figure A2), including the following:
  o the radius of the follicle shaft, $r_{0}$,
  o the radius of the follicle orifice, $r_{1}$,
  o the length of the follicle, $L_{1}$,
  o the number of follicles per unit area of skin surface, $N_{f}$, and
  o the proportion of follicles that are open.
- the diffusivity of the permeant within the fluid that occupies the infundibulum, $D_{inf}$. Here, the fluid is assumed to be sebum in the in vivo context. Under in vitro conditions, the fluid is assumed to be identical to the vehicle.
- the partition coefficient of the permeant in the infundibulum fluid with respect to water, $K_{inf/w}$.

We define infundibular permeability as $P_{inf/w}=f_{open}N_{f}K_{inf/w}D_{inf}/L_{1}$.

The lateral movement of the permeant between the follicle pathway and the skin is governed by a mass transfer coefficient $k_{ors}$ which depends primarily on an aggregate permeability that is evaluated from the skin and infundibulum dimensions, the stratum corneum permeability $P_{sc/w}$, and the viable epidermis permeability $P_{ed/w}$, as shown in Figure A3. The reader is referred to [13] and its accompanying supplementary materials for full mathematical details of the model.

### Appendix A.2. Pathway B—Transcellular pathway of Yu et al., 2021 [13]

Reference [12] adds a pathway through which hydrophilic solutes may penetrate through micropores and deformities within the lipid bilayers of the SC. This modification essentially modifies the resistance to permeant flow across the SC by adding a parallel resistance to the lipid bilayers that is more admissible with respect to hydrophilic permeants than are the SC lipids (Figure A4).

It is assumed in reference [12] that corneocytes are enveloped by seven lipid bilayers, each of permeability $k_{trans}$, which, in turn, is a QSPR that is a function of molecular weight. The combined resistance $R_{lip}$ (the inverse of permeability) of the seven stacked bilayers with respect to an adjacent water phase is given by

$$R_{lip}=\frac{7}{k_{trans}K_{lip/w}}.$$

The resistance (inverse permeability) via an aqueous micropore of a length that spans seven lipid bilayers is given by

$$R_{por}=\frac{7\delta }{\varepsilon_{3}{H(\lambda_{3})D}_{aq}}.$$

Here, quantity $D_{aq}$ is the diffusivity of the permeant in the aqueous micropore, and the dimensionless product $\varepsilon_{3}H(\lambda_{3})$ scales down the diffusivity. The factor, $\varepsilon_{3}$ is a non-dimensional scaling representing the degree of porosity of the lipid bilayers, $\lambda_{3}$ is the ratio of the permeant radius to the micropore radius, and $H\left(\lambda_{3}\right)$ quantifies the hindrance of the pores with respect to the flow of the permeant as a function of the ratio $\lambda_{3}$.

Since the permeating molecule may bypass the lipid bilayers via the porous route, the net resistance of the lipid bilayers can be approximated by the inverse of the sum of their permeabilities, $\frac{1}{\frac{1}{R_{lip}}+\frac{1}{R_{por}}}$.

The lipid bilayers envelope corneocytes, which are assumed to have a thickness t, a diffusivity $D_{cor}$, and a partition coefficient with respect to water $K_{cor/w}$. The resistance (inverse permeability) to permeant flow across a single corneocyte is given by

$$R_{int}=\frac{t}{D_{cor}K_{cor/w}}.$$

The average pathway across the stratum corneum can be viewed as a series of corneocytes of thickness t interspersed by a series of (on average) seven lipid bilayers, each of thickness δ. This pattern is therefore repeated $h_{sc}/m_{y}$ times, where $m_{y}=t+7\delta$. The net resistance, $R_{sc}$, across the stratum corneum is given by the combined series resistance of each unit of this repeating pattern:

$$R_{sc}=\frac{h_{sc}}{m_{y}}\left(R_{int}+\frac{1}{\frac{1}{R_{lip}}+\frac{1}{R_{por}}}\right)=\frac{h_{sc}}{m_{y}}\left(\frac{t}{D_{cor}K_{cor/w}}+\frac{1}{\frac{k_{trans}K_{lip/w}}{7}+\frac{\varepsilon_{3}D_{aq}H(\lambda_{3})}{7\delta }}\right)$$

This gives a stratum corneum permeability of $1/R_{sc}$.

If we define $\alpha =7\delta /m_{y}$, $R_{sc}$ can be re-written as

$$R_{sc}=h_{sc}\left(\left(1-\alpha \right)\frac{1}{D_{cor}K_{cor/w}}+\alpha \frac{1}{k_{trans}\delta K_{lip/w}}\frac{1}{1+\frac{\varepsilon_{3}D_{aq}H\left(\lambda_{3}\right)}{k_{trans}\delta K_{lip/w}}}\right)$$

Here, $\frac{\varepsilon_{3}D_{aq}H\left(\lambda_{2}\right)}{k_{trans}\delta K_{lip/w}}$ represents the ratio of permeability across the aqueous micropores to the permeability across lipid bilayers. When this ratio is much larger than unity, the overall transcellular permeability is mostly determined by the permeability through the micropores and takes on a value $P^{ion}$, where

$$P^{ion}=\frac{1}{h_{sc}}{\left(\left(1-\alpha \right)\frac{1}{D_{cor}K_{cor/w}}+\alpha \frac{1}{\varepsilon_{3}D_{aq}H\left(\lambda_{3}\right)}\right)}^{-1}$$

Otherwise, the permeability is approximated by that across the lipid bilayers and is denoted as $P_{tot}^{non}$:

$$P^{non}=\frac{1}{h_{sc}}{\left(\left(1-\alpha \right)\frac{1}{D_{cor}K_{cor/w}}+\alpha \frac{1}{k_{trans}\delta K_{lip/w}}\right)}^{-1}$$

Reference [12] then defines an aggregate permeability across the stratum corneum that is obtained from a weighted sum of these two values, given by

$$P_{sc/w}=P^{non}+\frac{1-f_{non/sc}}{f_{non/sc}}\;P^{ion}$$

Here, $f_{non/sc}$ denotes the fraction of the permeant that is non-ionized in the stratum corneum, which in turn depends on the stratum corneum pH and the permeant’s pK_a_, according to the Henderson–Hasselbalch relation.

Approximation of aggregate permeability *P*_(*tot*/*w*)_

We denote by $A_{tot}$ the area of skin to which a topical formulation is applied and let $A_{tot}=A_{skin}+A_{inf}$, where $A_{inf}$ is the infundibular cross-sectional area. For simplicity, we assume that the infundibulum runs parallel to the entire thickness of the skin membrane, which holds true in the case of the dermatomed skin experiments considered in this study. The aggregate permeability of the stratum corneum is given by

$$P_{tot/w}^{sc}=\frac{A_{skin}D_{sc}K_{sc}+A_{inf}K_{inf/w}D_{inf}\;\;}{h_{sc}\cdot \left(A_{skin}+A_{inf}\right)}$$

We similarly define the aggregate permeabilities across the epidermis and dermis, respectively, as

$$P_{tot/w}^{ed}=\frac{A_{skin}D_{ed}K_{ed}+A_{inf}K_{inf/w}D_{inf}\;\;}{h_{ed}\cdot \left(A_{skin}+A_{inf}\right)}$$

and

$$P_{tot/w}^{de}=\frac{A_{skin}D_{de}K_{de}+A_{inf}K_{inf/w}D_{inf}}{h_{de}\cdot \left(A_{skin}+A_{inf}\right)}$$

The aggregate permeability across the skin membrane is therefore given by

$$P_{tot/w}={\left(\frac{1}{P_{tot/w}^{sc}}+\frac{1}{P_{tot/w}^{ed}}+\frac{1}{P_{tot/w}^{de}}\right)}^{-1}.$$

It should be noted that this approximation does not account for the lateral permeability between the infundibulum and skin.

## References

[1] Praca F.S.G., Medina W.S.G., Eloy J.O., Petrilli R., Campos P.M., Ascenso A., Bentley M. (2018). Evaluation of critical parameters for in vitro skin permeation and penetration studies using animal skin models. Eur. J. Pharm. Sci..

[2] Feldmann R.J., Maibach H.I. (1969). Percutaneous penetration of steroids in man. J. Investig. Dermatol..

[3] Matta M.K., Florian J., Zusterzeel R., Pilli N.R., Patel V., Volpe D.A., Yang Y., Oh L., Bashaw E., Zineh I. (2020). Effect of Sunscreen Application on Plasma Concentration of Sunscreen Active Ingredients: A Randomized Clinical Trial. JAMA.

[4] Matta M.K., Zusterzeel R., Pilli N.R., Patel V., Volpe D.A., Florian J., Oh L., Bashaw E., Zineh I., Sanabria C. (2019). Effect of Sunscreen Application Under Maximal Use Conditions on Plasma Concentration of Sunscreen Active Ingredients: A Randomized Clinical Trial. JAMA.

[5] Mitragotri S., Anissimov Y.G., Bunge A.L., Frasch H.F., Guy R.H., Hadgraft J., Kasting G.B., Lane M.E., Roberts M.S. (2011). Mathematical models of skin permeability: An overview. Int. J. Pharm..

[6] Cleek R.L., Bunge A.L. (1993). A new method for estimating dermal absorption from chemical exposure. 1. General approach. Pharm. Res..

[7] Potts R.O., Guy R.H. (1992). Predicting skin permeability. Pharm. Res..

[8] Wilschut A., ten Berge W.F., Robinson P.J., McKone T.E. (1995). Estimating skin permeation. The validation of five mathematical skin permeation models. Chemosphere.

[9] Baba H., Ueno Y., Hashida M., Yamashita F. (2017). Quantitative prediction of ionization effect on human skin permeability. Int. J. Pharm..

[10] Dancik Y., Miller M.A., Jaworska J., Kasting G.B. (2013). Design and performance of a spreadsheet-based model for estimating bioavailability of chemicals from dermal exposure. Adv. Drug Deliv. Rev..

[11] Hamadeh A., Sevestre M., Edginton A. Implementation of Dancik et al (2013) Skin Permeation Model in MoBi2019. https://github.com/Open-Systems-Pharmacology/Skin-permeation-model.

[12] Kasting G.B., Miller M.A., LaCount T.D., Jaworska J. (2019). A Composite Model for the Transport of Hydrophilic and Lipophilic Compounds Across the Skin: Steady-State Behavior. J. Pharm. Sci..

[13] Yu F., Tonnis K., Kasting G.B., Jaworska J. (2021). Computer Simulation of Skin Permeability of Hydrophobic and Hydrophilic Chemicals—Influence of Follicular Pathway. J. Pharm. Sci..

[14] Blume U., Ferracin J., Verschoore M., Czernielewski J.M., Schaefer H. (1991). Physiology of the vellus hair follicle: Hair growth and sebum excretion. Br. J. Dermatol..

[15] Bouabbache S., Pouradier F., Panhard S., Chaffiotte C., Loussouarn G. (2019). Exploring some characteristics (density, anagen ratio, growth rate) of human body hairs. Variations with skin sites, gender and ethnics. Int. J. Cosmet. Sci..

[16] Otberg N., Richter H., Schaefer H., Blume-Peytavi U., Sterry W., Lademann J. (2004). Variations of hair follicle size and distribution in different body sites. J. Investig. Dermatol..

[17] Pagnoni A., Kligman A.M., el Gammal S., Stoudemayer T. (1994). Determination of density of follicles on various regions of the face by cyanoacrylate biopsy: Correlation with sebum output. Br. J. Dermatol..

[18] Seago S.V., Ebling F.J. (1985). The hair cycle on the human thigh and upper arm. Br. J. Dermatol..

[19] Wang T.F., Kasting G.B., Nitsche J.M. (2007). A multiphase microscopic diffusion model for stratum corneum permeability. II. Estimation of physicochemical parameters, and application to a large permeability database. J. Pharm. Sci..

[20] Chen L., Han L., Lian G. (2013). Recent advances in predicting skin permeability of hydrophilic solutes. Adv. Drug Deliv. Rev..

[21] Hamadeh A., Troutman J., Najjar A., Edginton A. (2022). A Mechanistic Bayesian Inferential Workflow for Estimation of In Vivo Skin Permeation from In Vitro Measurements. J. Pharm. Sci..

[22] Roy S.D., Flynn G.L. (1990). Transdermal delivery of narcotic analgesics: pH, anatomical, and subject influences on cutaneous permeability of fentanyl and sufentanil. Pharm. Res..

[23] Morris M. (1991). Factorial Sampling Plans for Preliminary Computational Experiments. Technometrics.

[24] Roy S.D., Flynn G.L. (1988). Solubility and related physicochemical properties of narcotic analgesics. Pharm. Res..

[25] Anderson B.D., Higuchi W.I., Raykar P.V. (1988). Heterogeneity effects on permeability-partition coefficient relationships in human stratum corneum. Pharm. Res..

[26] Nitsche J.M., Wang T.F., Kasting G.B. (2006). A two-phase analysis of solute partitioning into the stratum corneum. J. Pharm. Sci..

[27] Yun Y.E., Calderon-Nieva D., Hamadeh A., Edginton A.N. (2022). Development and Evaluation of an In Silico Dermal Absorption Model Relevant for Children. Pharmaceutics.

[28] Ya-Xian Z., Suetake T., Tagami H. (1999). Number of cell layers of the stratum corneum in normal skin—Relationship to the anatomical location on the body, age, sex and physical parameters. Arch. Dermatol. Res..

[29] Andrews S.N., Jeong E., Prausnitz M.R. (2013). Transdermal delivery of molecules is limited by full epidermis, not just stratum corneum. Pharm. Res..

